# From low beginnings to fluctuating trajectories: exploring motivational change in Thai learning

**DOI:** 10.3389/fpsyg.2026.1777762

**Published:** 2026-03-16

**Authors:** Xiaobin Ren, Min Zhang, Shutian Zhang

**Affiliations:** 1 School of Foreign Languages, Guangxi University, Nanning, China; 2 Asia-Pacific (Southeast Asia) Institute for Translation and Intercultural Studies, Guangxi University, Nanning, China

**Keywords:** foreign language learning, less commonly taught languages, motivational trajectories, Thai learning, Thai majors

## Abstract

This study investigated the evolving trajectories and influencing factors of Thai majors' language learning motivation in Chinese universities. Drawing on retrospective motivational mapping and semi-structured interviews with 21 participants, the study employed grounded theory to analyze motivational changes across 4 years of undergraduate study. The findings revealed that students' motivation was characterized by sharp fluctuations, early-stage rises from low baselines, a significant decline during the sophomore-to-junior transition, and erratic shifts in the final year. Despite some common trends, individual motivational paths displayed considerable diversity. Grounded theory analysis further identified four interconnected systems—macro, exo, proximal, and habitual—that collectively shaped learners' motivational dynamics. Notably, many Thai majors entered their programs with relatively low initial motivation, contrasting with findings from studies on major world languages. In addition, learners' motivation was highly sensitive to macro-level influences such as political events, and overseas exchange experiences did not yield the motivational gains commonly reported in Western study-abroad contexts. The study makes two key contributions: theoretically, it extends the explanatory scope of motivation research to less commonly taught languages by constructing a visualized, multi-layered model within the framework of complex dynamic systems theory; practically, it provides insights for curriculum design, student support, and policy development by highlighting critical phases and contextual influences on motivational sustainability.

## 1 Introduction

Thailand's strategic location and economic significance in the Asia Pacific region have positioned the country as a key economic hub within the Association of Southeast Asian Nations (ASEAN), enabling it to play a crucial role in regional trade, manufacturing, and global supply chains (Wongwuttiwat and Lawanna, 2018). As an official ASEAN language, Thai has garnered increasing global interest as a foreign language (Li, 2023; Watthanapas et al., 2023). Given Thailand's growing international influence (Yuenyong and Chaipiboolwong, 2022), the ability to communicate effectively in Thai has become increasingly valuable for those seeking to engage with the country's expanding presence in regional and global affairs, thereby raising important questions about what drives learners to pursue and sustain Thai language study. Investigating the learning motivation of university students majoring in Thai is therefore essential for fostering cross-cultural communication, strengthening international trade and tourism collaborations, and deepening mutual understanding in an increasingly interconnected world.

Existing research on foreign language learning motivation has predominantly examined external influences such as teaching methods (Afzali and Izadpanah, 2021; Popovska Nalevska and Kuzmanovska, 2020; Somers and Llinares, 2021), curriculum content (Banegas, 2019; Zhang et al., 2020), school environment (Henry and Thorsen, 2018; Jong, 2020), and sociocultural factors (Chen et al., 2005; Kristiawan et al., 2022). Additionally, studies have explored intrinsic demographic factors, including gender, age, and grade level (Carreira, 2011; Yashima et al., 2017). However, much of this research has adopted a static, cross-sectional perspective, offering limited insight into the evolution and dynamic nature of language learning motivation over time (Dewaele and Meftah, 2024). Furthermore, existing studies have predominantly focused on widely spoken languages such as English and Spanish, while research on the learning motivation of less commonly taught languages, such as Thai, remains scarce. Addressing these gaps is crucial for broadening our understanding of how language learning motivation fluctuates and adapts in response to various internal and external influences.

To this end, the present study examines the evolving learning motivation of 21 university students majoring in Thai, employing a qualitative multi-method approach that integrates motivational retrospective mapping and interviews. By identifying the characteristics and underlying causes of motivational changes, this study seeks to contribute to both theoretical and practical discussions on language learning motivation. The findings may offer valuable implications for refining teaching strategies, informing educational policies, and ultimately enhancing Thai language education, thereby promoting broader linguistic and cultural exchange on a global scale.

## 2 Literature review

### 2.1 Studies on foreign language learning motivation

Motivation has long been recognized as a key determinant in foreign language (L2) learning (Dörnyei, 1998). Gardner and Lambert (1959) were pioneers in this field, identifying two main types of motivation: integrative motivation, which reflects a learner's desire to connect with the target language community, and instrumental motivation, which highlights practical benefits like career advancement. Their research established a foundation for decades of L2 motivation studies, initially focusing on these relatively stable, trait-like forms of motivation. Over time, scholars expanded these ideas by integrating cognitive psychology and socio-educational frameworks (Hatoss, 2008). While early studies laid a foundation for understanding motivation, they largely employed static, trait-based models that overlooked the fluid and context-dependent nature of learners' experiences (Dörnyei et al., 2015).

More recent research on foreign language learning motivation has predominantly relied on cross-sectional approaches, focusing on specific external or demographic factors without tracking changes over time. For instance, Afzali and Izadpanah (2021) found that the flipped classroom model enhanced learner motivation but did not conduct follow-up assessments. Henry and Thorsen (2018) highlighted the positive influence of teacher-student relationships on motivation but relied on one-time observations and interviews. Similarly, Yashima et al. (2017) examined gender differences in motivation using a single round of questionnaires, while Carreira (2011) analyzed motivation in relation to age and grade level through two isolated surveys. These studies provide valuable insights but fail to capture the dynamic fluctuations in motivation that occur as learners navigate challenges and achievements. Given that motivation is not a fixed trait but rather a complex, evolving process, understanding its temporal variations is essential (Fu and Chen, 2025; Teng, 2025). A longitudinal perspective can offer deeper insights into how motivation develops, sustains, or declines over time, providing a more comprehensive basis for effective pedagogical interventions. Therefore, there is a pressing need for research that explores the dynamic nature of foreign language learning motivation through longitudinal or retrospective methodologies.

In addition, most existing studies on foreign language learning motivation focus on widely spoken languages such as English and Spanish. For example, Nagle (2018) examined motivation in relation to Spanish pronunciation improvement, while Carreira (2011) documented declining English learning motivation among Japanese students. While research on language learning motivation has extensively covered widely spoken languages, studies on Southeast Asian languages, particularly Thai, remain scarce. This gap is particularly noteworthy given the growing global interest in Thailand's economic, cultural, and geopolitical presence, which has led to an increasing number of learners pursuing Thai as a foreign language. Therefore, investigating the motivation to learn Thai can not only enrich the broader field of language learning motivation by diversifying language contexts, but also generate practical insights for curriculum design, teacher training, and learner support tailored to less commonly taught languages.

### 2.2 Thai education research

Currently, some research has examined Thai education, but these studies primarily focus on native speakers or heritage learners rather than foreign language learners. For instance, Durongkaveroj (2023) identified significant challenges in Thai language education for native learners, including low proficiency outcomes, regional disparities, and the need for curriculum reform. Similarly, Chantarat and Chookhampaeng (2023) and Chanthao (2018) explored Thai language instruction in primary schools in Thailand, emphasizing challenges faced by native Thai children. In addition, studies on heritage language learning, such as Jamcharoensup (2014) on Thai instruction in Swiss Buddhist Sunday schools, have focused on cultural and familial dynamics in language retention rather than foreign language acquisition. While these studies provide valuable insights into language maintenance among native and heritage speakers, they do not address the experiences of foreign learners studying Thai in a non-immersion context.

In the Chinese context, where Thai education has been expanding steadily, existing research still tends to concentrate on phonological and lexical aspects, with limited attention to the lived experiences and motivational dynamics of Thai language learners. For example, Li (2025) examined the international variants of the modal particle *na* in Thai and their role in expressing intersubjectivity in natural discourse, while Li (2023) investigated the diachronic and synchronic evolution of the Chinese loanword *Guo (*过*)* into Thai, focusing on its phonological form and grammaticalization process. These studies, though valuable for understanding Thai linguistic features, reflect the predominant focus on structural aspects of the language, rather than the perspectives, needs, or motivations of Thai language learners themselves.

Understanding the motivation behind learning Thai as a foreign language is crucial, as foreign-language learners face distinct challenges and learning environments compared to native speakers (Butzkamm, 2003). Investigating these motivational dynamics can provide deeper insights into the incentives and barriers learners encounter, ultimately informing the development of more tailored pedagogical approaches. Given the increasing global interest in Thai language and culture, research in this area is essential for fostering more effective language learning experiences and expanding cross-cultural understanding.

### 2.3 Complex dynamic systems theory

Complex Dynamic Systems Theory (CDST) is a holistic approach for analyzing complex systems across various phenomena (Van Geert, 2008), aiming to understand dynamic interactions of processes and elements driving events and changes (Van Geert and Van Dijk, 2002). Originally from the natural sciences, CDST has been applied to explain complex systems in fields like biology, physics, chemistry, and mathematics (Amerstorfer, 2020). Larsen-Freeman (1997) introduced CDST into the field of applied linguistics, adopting a comprehensive perspective that views events as dynamic and complex rather than predictable, static, isolated, or in a straightforward cause-and-effect relationship. Based on CDST, current language learning motivation research has evolved into a phase recognizing learning as a highly personalized, variable process, challenging to be predicted due to factors like context, sociocultural constraints, and interactions with peers and teachers (Castro, 2018).

The application of CDST in second and foreign language motivation research can be observed in three key areas. First, it has contributed to theoretical advancements by reframing motivation as a dynamic, evolving process rather than a static attribute (Al-Hoorie and MacIntyre, 2019; Dörnyei et al., 2016). This perspective aligns with our study's goal of examining Thai language learning motivation as an ongoing, context-sensitive phenomenon rather than a fixed state. Second, CDST has inspired new methodological approaches, such as Retrodictive Qualitative Modeling (Dörnyei, 2014), which aim to trace the developmental trajectory of motivation by analyzing the factors that lead to particular motivational states. Our study follows this line of research by adopting a longitudinal approach to capture fluctuations in learners' motivation over time. Third, CDST has been used to explain the driving forces behind motivational changes, with studies demonstrating how motivation emerges from the interaction between personal goals, institutional settings, and broader sociocultural influences (Dörnyei et al., 2015). This perspective is particularly relevant to our research, as we seek to uncover the key factors influencing Thai language learning motivation among Chinese university students.

Despite the growing application of CDST in second language acquisition research, most studies have focused on English and other widely spoken languages, leaving languages such as Thai underexplored. Given that motivation in learning Thai as a foreign language is influenced by unique sociocultural and institutional factors, a CDST perspective allows us to examine how individual learners' motivation evolves through dynamic interactions with their learning environment. By employing CDST, this study seeks to address two key research questions:

1. What are the characteristics of changes in the language learning motivation of college Thai majors?

2. What are the factors causing the changes in language learning motivation among college Thai majors?

## 3 Research design

### 3.1 Research background

Compared with major world languages such as English, Japanese, or Spanish, Southeast Asian languages, including Thai, are learned by a much smaller population in China, and are offered by a limited number of higher education institutions. Thai language programs are primarily concentrated in universities located in Southwest China and in specialized foreign language universities. This distribution is largely shaped by geographical proximity and regional engagement, as Southwest China maintains closer economic, cultural, and educational ties with Southeast Asia, particularly Thailand. As a result, universities in this region have become the main institutional sites for Thai language education. In addition, similar to other foreign language majors in China, Thai programs are characterized by a strong gender imbalance, with female students substantially outnumbering male students.

### 3.2 Participants

A theoretical sampling approach (Corbin and Strauss, 2014) was employed in this study to select participants who could provide rich, diverse, and meaningful insights into their motivational changes in learning Thai. Given the qualitative research goal of exploring the depth and complexity of participants' experiences, the sampling process was designed to capture a wide range of perspectives and backgrounds.

The participant selection was guided by the following criteria: (1) Enrollment in a Thai major program to ensure relevance to the study focus. (2) Representation across multiple universities to ensure institutional diversity. (3) Selection of students from universities in different geographic regions to capture diverse socio-cultural and educational contexts. (4) At least 3.5 years of formal Thai language learning experience to ensure participants had substantial exposure to the language and its learning process.

In total, 21 undergraduate students from eight universities across China participated in this study. The sample included students from comprehensive universities, normal universities, foreign language universities, and private institutions, ensuring a diverse range of institutional contexts. Participants were selected from different geographic regions, with the majority studying in universities located in Southwest China, which shares closer geographical and cultural ties with Thailand.

Detailed demographic and background information on the participants is provided in Table 1.

**Table 1:** Demographic information of interviewees.

**Demographics**	**Category**	***F***	**%**
Gender	Male	4	19.05
	Female	17	80.95
Age	21	4	19.05
	22	13	61.9
	23	3	14.29
	24	1	4.76
Institution type	Comprehensive	11	52.38
	Normal (Teachers')	1	4.76
	Minzu (Ethnic)	4	19.05
	Foreign/International studies	4	19.05
	Private	1	4.76
Location	Northwest China	2	9.52
	North China	2	9.52
	Southwest China	17	80.96

### 3.3 Instruments

This study employed two qualitative research instruments—motivational retrospective mapping and semi-structured interviews—to comprehensively explore the motivational changes of college students majoring in Thai. Prior to the interviews, participants were first asked to reflect on their Thai language learning experiences and visually plot their motivational trajectories using a retrospective mapping technique. This process allowed participants to systematically recall fluctuations in their motivation over time, providing a structured foundation for the subsequent semi-structured interviews, where they elaborated on the reasons behind these motivational shifts. By integrating these two methods, the study effectively captured both the dynamic nature of motivation over time and the underlying factors influencing its evolution.

#### 3.3.1 Motivational retrospective mapping

Drawing on Dörnyei (2014)'s retrospective qualitative modeling method, this study employed motivational retrospective mapping to visually capture changes in students' motivation for learning Thai over the course of their college years. A total of 21 participants were asked to reflect on and plot their motivational trajectories, providing a visual representation of fluctuations in their motivation levels throughout their academic journey.

This approach is supported by prior research on motivational dynamics. For instance, Wang and Littlewood (2021), as well as Song and Kim (2017), employed this motivational retrospective mapping in their studies on the dynamics of students' demotivation, and Ren and Zhou (2022) also used this approach when investigating the research motivation of university English teachers. These studies underscore the effectiveness of retrospective mapping in illustrating the dynamic and individualized nature of learners' motivation over time.

**Figure 1:**
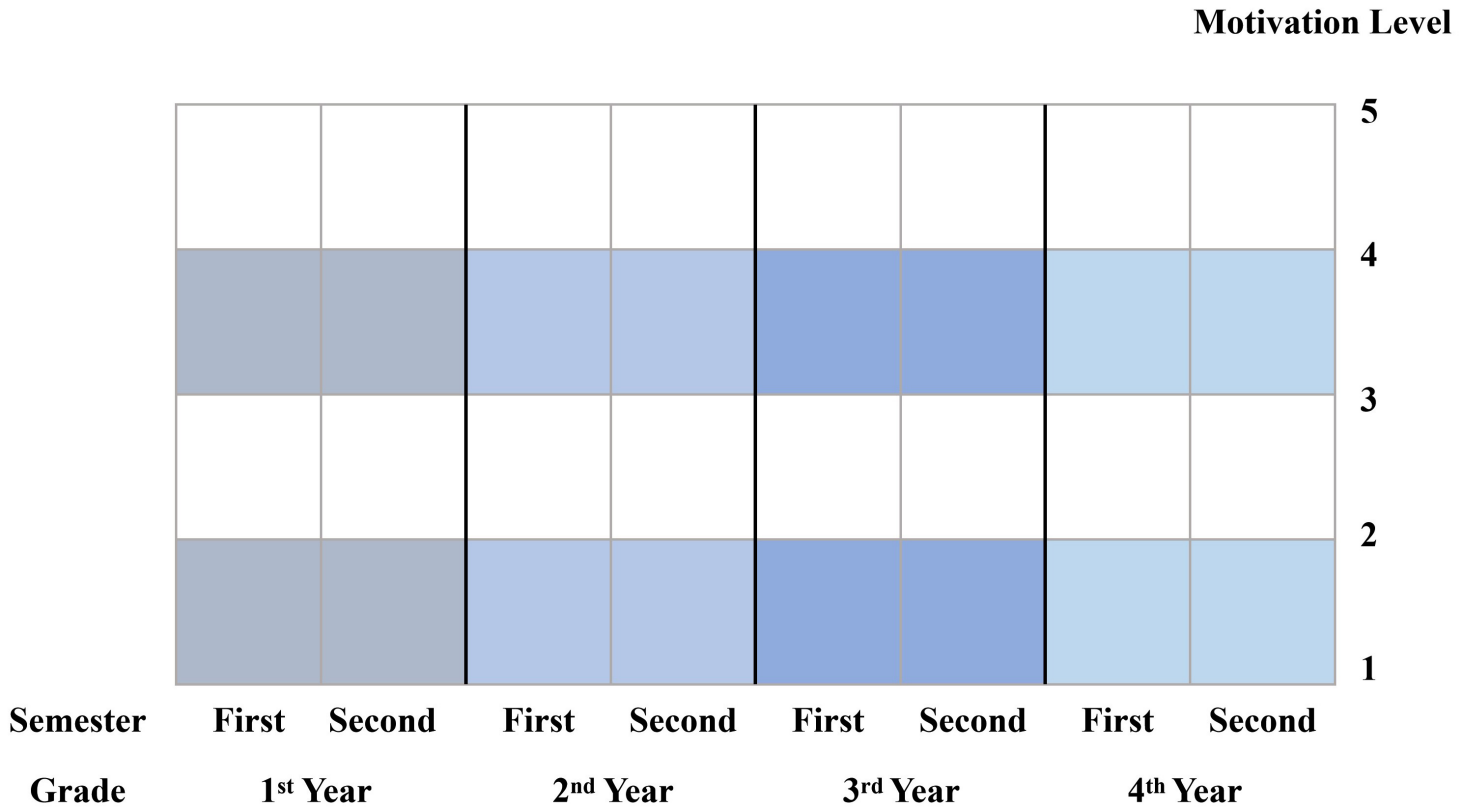
Motivational retrospective mapping.

Following the approach of Ren et al. (2024), motivation in this study was categorized into five levels, ranging from 1 (lowest motivation) to 5 (highest motivation). The temporal scope covered the entire 4 year undergraduate period, with specific reference points at each semester. Based on this structure, participants were asked to retrospectively chart their motivational trajectories, and the template for recording these fluctuations is presented in Figure 1.

Motivational retrospective mapping is a useful tool for capturing long-term motivational change, but it also involves potential validity threats, including recall bias, anchoring effects, and comparability across participants. To enhance trustworthiness, several procedural strategies were adopted. Participants were provided with temporal scaffolds and anchoring prompts, such as major academic milestones, examinations, overseas exchange experiences, and disruptions caused by the COVID-19 pandemic, which served as cognitive anchors to facilitate more structured recall. During the interviews, mapped trajectories were revisited and participants were asked to explain major fluctuations, allowing for cross-checking and clarification.

To improve calibration and comparability of the five-level motivation scale, all participants were given a shared frame of reference prior to the task, with explicit explanations and concrete examples for each level, followed by a brief trial run. Importantly, the mapped motivation levels should not be treated as objective longitudinal measurements, but rather as a semi-quantified visual approximation of participant' subjective perceptions. The numerical values function as heuristic devices for identifying patterns of change, and the mapping results are therefore interpreted in conjunction with qualitative interview data, with causal claims avoided.

#### 3.3.2 Interviews

Semi-structured interviews were utilized as the primary research tool, with one-on-one interviews conducted with participants. To ensure the reliability and validity of the interviews, a semi-structured interview outline was developed in advance. The initial version of the outline was reviewed by an experienced foreign language teacher holding a doctoral degree, who evaluated the questions in terms of their relevance to the research questions, clarity of wording, and overall logical flow. Based on this feedback, several items were refined to improve conceptual alignment and comprehensibility. In addition, the interview guide was piloted with two Thai majors prior to formal data collection, which led to minor revisions in question phrasing and sequencing. These procedures helped enhance the content validity and practical suitability of the interview instrument.

A total of 21 interviews were conducted, with an average duration of approximately 38.7 min per interview. The interview questions primarily focused on the changes in motivation among Chinese university students majoring in Thai and the factors contributing to these changes. During the interviews, participants were asked questions such as “Can you recall any specific events or turning points that significantly affected your motivation?” and “How did your motivation to learn Thai change from your freshman year to your senior year?” These questions were designed to prompt detailed recollections of motivational shifts and to explore the contextual and personal factors that shaped these changes over time.

Before the interviews commenced, participants were introduced to the research objectives. They were assured that all collected data would be kept confidential and used solely for research purposes. Additionally, informed consent was obtained from all participants, with explicit permission granted for recording the interviews.

### 3.4 Data analysis

The data analysis process began with transcribing the interview recordings into textual data, followed by data cleaning to ensure that the transcriptions accurately reflected the recorded content. The cleaned transcripts were then imported into Nvivo 14 for systematic coding and analysis.

To analyze the qualitative data, this study adopted a grounded theory approach (Corbin and Strauss, 2014), which is particularly well-suited for uncovering the underlying causes of complex, context-sensitive phenomena. Rather than testing predefined hypotheses, grounded theory emphasizes the inductive generation of theory from empirical data, making it especially appropriate for exploring the multifaceted reasons behind motivational changes among Thai majors and for constructing a data-driven explanatory model (Corbin and Strauss, 2014). Given the limited existing research on this topic, grounded theory provided a flexible yet rigorous methodological framework that allowed themes and patterns to emerge from participants' lived experiences without being constrained by prior theoretical assumptions.

Following the procedures proposed by Corbin and Strauss (2014), the coding process involved three stages: open coding, axial coding, and selective coding. These stages were used to progressively generate initial concepts, integrate them into categories, and construct an explanatory theoretical model. Two researchers were involved in the coding process. Both coders had prior training in qualitative research methods and grounded theory analysis. The initial coding was conducted independently, after which the two coders compared their coding results and discussed discrepancies until consensus was reached. This collaborative process helped reduce individual bias and enhance the reliability of category construction.

To enhance the trustworthiness of the analysis, several strategies were adopted. First, triangulation was achieved by integrating data from motivational retrospective mapping and semi-structured interviews. Second, peer debriefing was conducted through discussions with two senior scholars in foreign language education, who reviewed the coding framework and theoretical model. Third, member checking was implemented by sharing preliminary interpretations with several participants to confirm the credibility of the findings. Finally, reflexivity was addressed by acknowledging the researchers' positionality. As researchers working within the Chinese higher education system and familiar with foreign language programs, we were aware of potential interpretive biases. To mitigate this, we continuously returned to the original data, engaged in team-based coding, and relied on external peer feedback to critically examine our assumptions and interpretations.

Data saturation was assessed in terms of meaning saturation rather than mere code saturation. By the 18th interview, no substantially new subcategories or conceptual relationships emerged, and subsequent interviews mainly served to refine and enrich existing categories, indicating that theoretical saturation had been achieved.

All interviews were conducted in Chinese, which was also the working language for transcription and data analysis. To facilitate understanding for international readers, selected interview excerpts presented in the Results section were translated into English. The initial translation was carried out by one of the authors, who is bilingual in Chinese and English, and subsequently proofread by a Master's student majoring in Translation Studies. Any discrepancies were discussed and resolved through comparison with the original Chinese transcripts to ensure semantic accuracy and conceptual equivalence.

## 4 Results

### 4.1 Motivational trajectories

To explore the longitudinal patterns of motivational change, this study employed retrospective motivational trajectory maps, in which each participant plotted their self-assessed motivation levels—ranging from 1 (lowest) to 5 (highest)—across the eight semesters of their undergraduate study. A total of 21 valid trajectory maps were collected.

**Figure 2:**
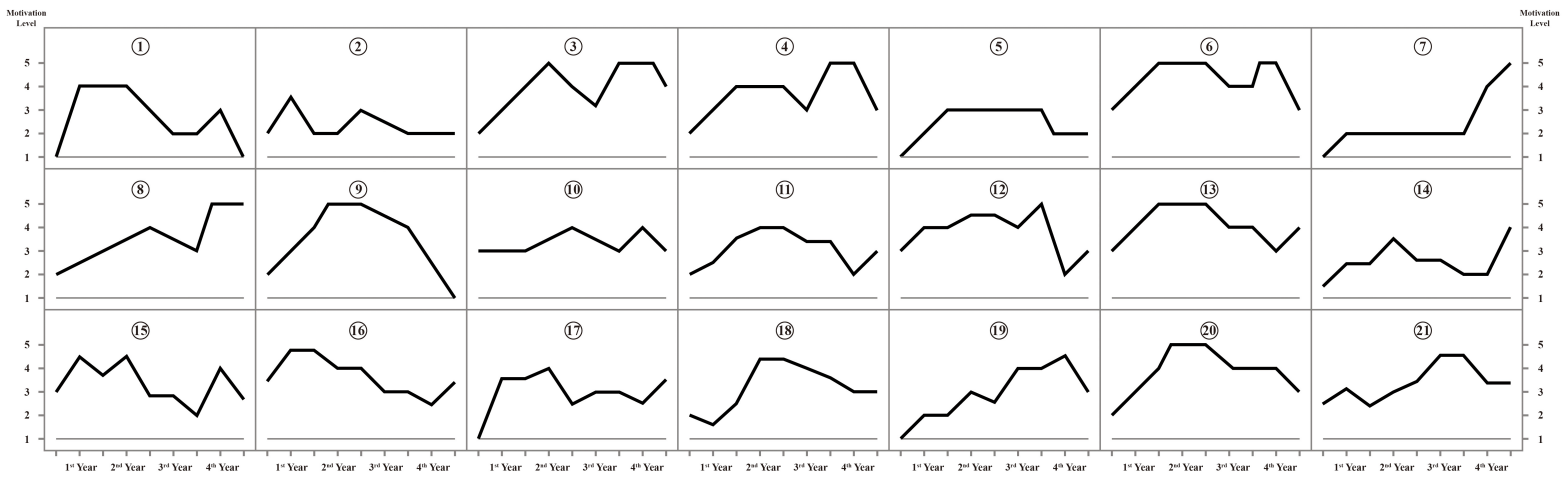
Motivation trajectories of 21 participants.

Instead of presenting these trajectories separately, the 21 individual motivational trajectories were organized into a small-multiples visualization (Figure 2), which allows each participant's motivational pattern to be displayed clearly while making it easier to identify overall developmental tendencies and recurrent patterns across the cohort within a single integrated figure. Based on an overall inspection of the 21 cases, several dominant developmental patterns were identified, including early-stage motivational rise (*n* = 19), a marked decline during the sophomore-to-junior transition (*n* = 14), and heightened volatility in the final year (*n* = 18).

### 4.2 Factors influencing motivational change

To further investigate the underlying causes of motivational change among Thai majors, this section presents the results of a grounded theory analysis based on 21 in-depth interviews. Following the grounded theory methodology, data were analyzed through three iterative coding phases: open coding, axial coding, and selective coding. These steps enabled the systematic identification, categorization, and integration of key themes related to the dynamics of learning motivation. Through this process, a multi-level explanatory model was developed to capture the complex interplay of external and internal factors that were perceived to shape fluctuations in students' motivation to learn Thai.

#### 4.2.1 Open coding

In grounded theory, open coding refers to the initial analytical process in which qualitative data—typically from interviews—is broken down into discrete units of meaning. These units were assigned conceptual labels (initial concepts) that captured the essence of participants' statements. Through constant comparison and iterative refinement, these concepts were then grouped into more abstract and inclusive subcategories that reflected shared patterns or themes across cases (Corbin and Strauss, 2014).

In this study, open coding was conducted using line-by-line analysis of 21 interview transcripts. A total of 69 initial concepts were identified, which were further clustered into 18 subcategories. For example, from the interviewee's statement “After the two countries signed new cooperation agreements, I felt learning Thai would give me better career chances,” we derived the initial concept “perceived increased value of Thai due to diplomatic agreements.” This was then grouped under the broader subcategory “Political events,” which includes various instances where geopolitical dynamics influence learners' motivation.

Due to space limitations, Table 2 presents a selection of 10 representative translated examples of the open coding process. Each example includes the original interview excerpt, the corresponding initial concept, and its assigned subcategory.

**Table 2:** Examples of open coding.

**Interview excerpt**	**Initial concepts**	**Subcategories**
“After the two countries signed new cooperation agreements, I felt learning Thai would give me better career chances.”	Perceived increased value of Thai due to diplomatic agreements	Political events
“I chose Thai because I heard there are more job opportunities in Southeast Asia now.”	Thai perceived as gateway to regional employment	Job market
“Our university changed the credit requirements for Thai majors, which made it harder to keep up.”	Increased academic pressure due to curriculum adjustment	Educational regulations
“My parents didn't support me learning Thai at first—they thought it was useless.”	Lack of familial support for Thai learning	Family environment
“When I heard that I might have a chance to go to Thailand on exchange, I suddenly became more motivated.”	Study abroad prospects stimulate motivation	Overseas opportunities
“Our Thai teacher was passionate and made the class very engaging, so I started to enjoy learning more.”	Positive classroom engagement through teacher enthusiasm	Teachers
“I used to feel I wasn't good at learning languages, so I doubted myself a lot.”	Low confidence in language learning ability	Self-efficacy
“I've always had a plan to work in international business, and Thai fits into that plan.”	Thai learning aligned with long-term career planning	Personal planning
“Honestly, I chose this major just because I liked Thai TV shows.”	Interest in Thai popular culture as entry point	Attitudes
“In my second year, I studied harder because I didn't want to fall behind others.”	Increased effort due to competitive pressure	Motivational intensity

#### 4.2.2 Axial coding

Axial coding is the second stage of grounded theory analysis, in which the initial concepts and subcategories generated through open coding are re-examined to identify conceptual relationships and organized into higher-level main categories (Corbin and Strauss, 2014). This process involves identifying causal conditions, contextual factors, and interconnections across subcategories, which enables the development of a more integrated theoretical framework.

Importantly, these higher-order categories were not pre-defined prior to analysis, but emerged inductively during axial coding as conceptual abstractions that captured recurring patterns across the 18 subcategories. In this study, 18 subcategories were further clustered into five overarching main categories. For example, the subcategories “Disruptive incidents,” “Political events,” and “Job market” were all coded under the main category “Macro system.” These three subcategories reflect external, large-scale sociopolitical and structural forces that participants perceived as shaping motivation in indirect but impactful ways. Disruptive incidents, such as the COVID-19 pandemic, were frequently described as sudden disruptions to learning formats and environments, often diminishing motivation temporarily or prompting reassessment of academic goals. Political events, such as bilateral diplomatic visits or agreements between China and Thailand, enhanced the perceived cultural and strategic value of learning Thai. Job market considerations, including employment prospects in Southeast Asia, were reported as informing students' long-term, goal-oriented motivations. These influences are macro-level in nature—external to the individual and institution—and justify their classification under the Macro system, as they operate at a national or global level beyond the learner's immediate control but significantly inform their perceived utility and direction of learning.

To ensure conceptual clarity and theoretical coherence, the five system-level categories were constructed based on explicit decision rules during axial coding. Specifically, categories were differentiated according to the level at which an influence primarily operates and the degree of proximity to learners' direct learning activities. This rule-based classification ensured that each subcategory was positioned according to its dominant functional role in shaping motivational change, rather than merely its thematic similarity. During axial coding, several subcategories initially exhibited ambiguous boundaries across systems. For example, Overseas opportunities could plausibly be interpreted as a macro-level factor, as national mobility policies and international relations shape access to study abroad. However, based on our decision rules, this subcategory was ultimately classified under the Exosystem, because participants' motivational experiences were primarily mediated through university-level arrangements (e.g., institutional exchange programs, credit recognition, and administrative selection processes) rather than direct engagement with national policy structures.

Table 3 presents how all 18 subcategories were grouped into five main categories, along with interpretive descriptions of each main category's scope.

**Table 3:** Axial coding results.

**Subcategories**	**Main categories**	**Interpretive definition of main category**
Disruptive incidents	Macro system	Broader sociopolitical and structural forces—such as global crises, diplomatic relations, or labor market trends—that influence learners' perceptions of the value, urgency, and utility of language learning.
Political events		
Job market		
Educational regulations	Exosystem	Institutional and family-level factors that indirectly shape students' learning environments and decisions.
Family environment		
Examinations		
Overseas opportunities		
Teachers	Proximal system	Immediate classroom-level conditions and interpersonal dynamics that directly affect learners' day-to-day engagement and academic motivation.
Peers		
Textbooks		
Emotional experience	Habitual system	Learners' internal dispositions, routines, and affective responses that persist over time and shape motivation from within.
Personal planning		
Self-efficacy		
Attitudes		
Motivational intensity	Motivation	Key attributes of the motivation construct itself, including its strength, orientation, temporal variation, and critical shifts over time.
Directionality of goals		
Temporal fluctuation		
Turning points		

#### 4.2.3 Selective coding

Selective coding is the final stage in grounded theory analysis, in which the researcher identifies a core category that integrates and explains the relationships among all other categories and subcategories developed during earlier coding stages (Corbin and Strauss, 2014). While open coding generates initial concepts and axial coding establishes conceptual groupings, selective coding aims to synthesize the data into a coherent theoretical framework that captures the central phenomenon under investigation.

In this study, the selective coding process began with a comparative analysis of the five main categories and their subcategories, with particular attention paid to their interconnections, causal relationships, and relevance to the research focus—motivational change among Thai majors. Through iterative reflection and constant comparison, the core category “Motivation dynamics” was identified. Beyond identifying “Motivation dynamics” as the core category, selective coding further aimed to explicate the interactional mechanisms among the different systems. Rather than treating each system as an independent layer, the analysis revealed that motivational change appeared to emerge through continuous cross-level processes, in which macro- and exosystem factors shaped learners' motivation primarily by restructuring proximal learning conditions, and were subsequently internalized into habitual dispositions. In this sense, motivational dynamics are not driven by isolated factors, but emerge from the dynamic coupling of sociopolitical contexts, institutional arrangements, classroom experiences, and learners' internal meaning-making processes.

At the macro–exosystem level, disruptive incidents were often reported to reshape oversea opportunities, thereby indirectly influencing students' motivation. For example, one participant explained how the COVID-19 pandemic affected overseas exchange opportunities: “Because of the pandemic, all exchange programs were suspended, and I lost the chance to go to Thailand. That made me feel my learning had no real direction anymore.” This suggests that participants perceived macro-level disruptions as constraining exosystem conditions such as international mobility.

At the exosystem-proximal level, institutional regulations further shaped classroom practices and learning resources. One student noted that educational policies limited textbook updates: “Our university has very strict procedures for changing textbooks, so we still use very old materials. Sometimes the content feels outdated and irrelevant,” suggesting that exosystem rules were perceived to influence proximal learning conditions.

At the proximal-habitual level, teaching styles were frequently internalized into learners' long-term attitudes. As one participant reflected, “Some teachers just read from the slides and never interact with us. After a while, I started to feel Thai was boring and meaningless.” Finally, habitual dispositions such as personal planning directly shaped motivational dynamics: “I realized I probably won't work in any Thai-related job in the future, so I don't feel it's necessary to put too much effort into learning it now.” Together, these accounts demonstrate a cascading process in which macro- and exosystem forces operate through proximal experiences, are internalized into habitual orientations, and ultimately reconfigure learners' motivational trajectories.

**Figure 3:**
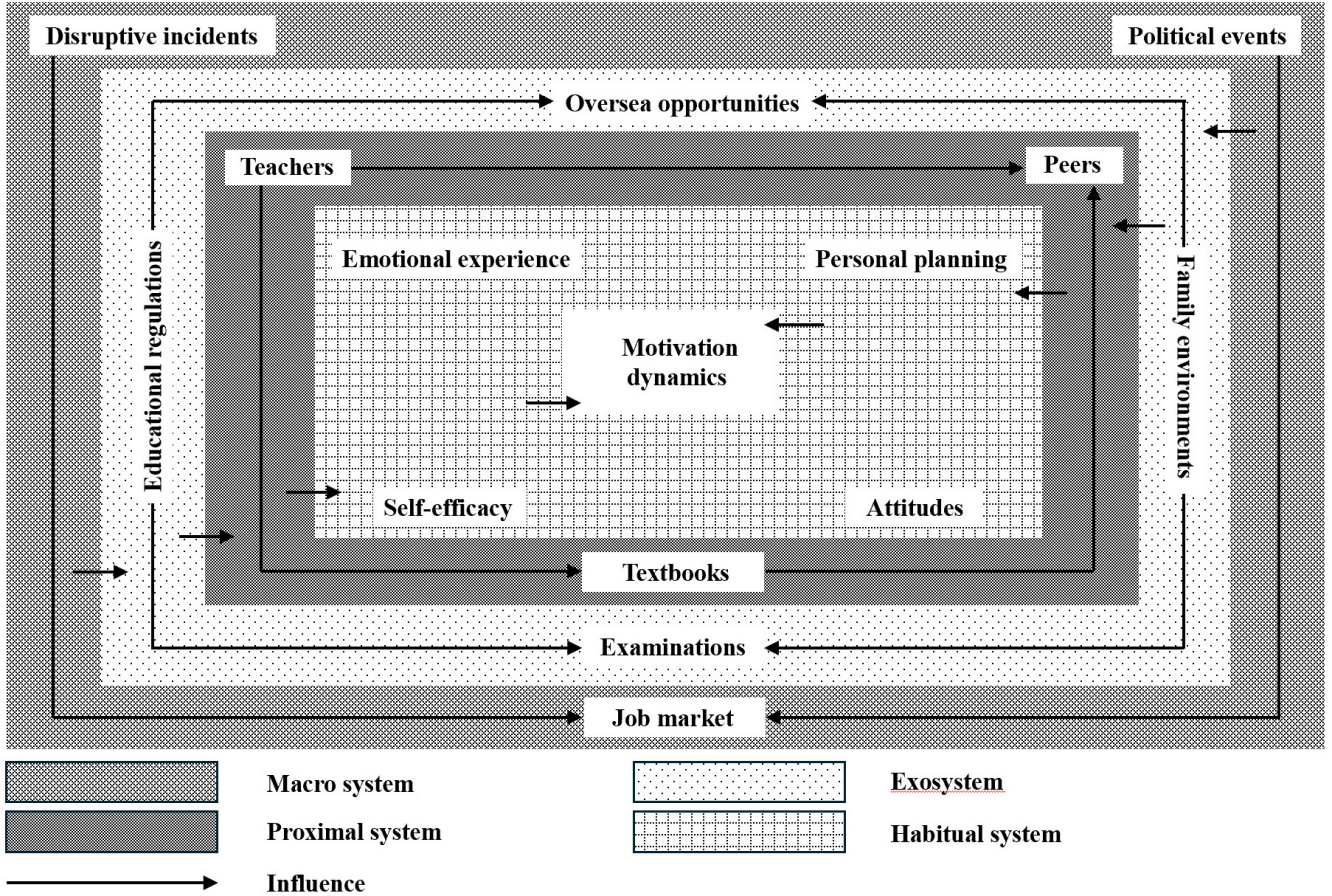
Theoretical model.

Figure 3 presents the integrative model developed through selective coding and explains how the core category is linked to each of the five main categories.

## 5 Discussion

### 5.1 Motivational trajectories

The motivational trajectories of Thai language learners in this study displayed both pronounced individual variability and non-linear fluctuation over time—two features that align closely with the principles of CDST (Larsen-Freeman and Cameron, 2008). Each participant followed a distinct path, shaped by diverse experiences and internal dynamics, while overall motivation levels rose and fell unpredictably across the three to four academic years. These findings echo patterns observed in studies on motivation in English (Waninge et al., 2014) and Spanish (Lu et al., 2019) learning contexts, suggesting that non-linearity and learner-specific trajectories may be consistent with patterns of foreign language motivation regardless of the target language. This reinforces the argument that motivation should be understood not as a stable trait but as a dynamic process, sensitive to context and interaction. Consequently, educators should move beyond static or one-size-fits-all approaches to motivation enhancement, and instead adopt flexible, adaptive strategies that help learners regulate and navigate motivational fluctuation throughout their language learning journey.

Many students began their Thai language programs with notably low motivation, which subsequently rose during the first year—a pattern that contrasts with findings in studies of major world languages such as German or English, where learners often exhibit strong initial enthusiasm (Busse and Walter, 2013; Castro, 2018), but aligns with recent research on Vietnamese language learners in China who showed similar early-stage motivational stagnation due to lack of interest or program reallocation (Ren and Wang, 2025a). This discrepancy may be attributed to the fact that a considerable number of students in Thai programs were not self-selected but admitted through administrative reassignment (Ren and Wang, 2025b), leading to limited intrinsic interest in the language. Additionally, some students held biased perceptions of Thailand's global status, viewing its economy and international influence as inferior to China's, which further undermined their initial motivation. As one participant remarked, “Thailand is just a small country, and its economy doesn't seem well developed, so at the beginning I couldn't see why learning Thai would matter.” This illustrates how macro-level sociocultural perceptions were described as contributing to learners' low initial motivation. From the perspective of CDST, this stage exemplifies sensitivity to initial conditions: learners' early motivational states are shaped by institutional factors and sociocultural perceptions. The motivational rise observed later in the first year reflects self-organizing processes, as students gradually adjusted to their new academic environments through peer interaction, curriculum engagement, and increasing cultural awareness. These findings underscore the importance of targeted support during the initial learning phase, aimed at transforming learner resistance into constructive engagement.

A pronounced decline in motivation during the sophomore to junior years was observed among most participants. This pattern corresponds to CDST's idea of a phase shift (Larsen-Freeman, 1997), in which the system transitions from a relatively stable state to one of decline or disruption. It may also signal a change in attractor states, as students' initial interests or goals lose salience. As one student reflected, “By the third year, many of the course contents felt repetitive, and much of it could be studied independently. What I really wanted was the chance to explore something new, but that was missing, so my motivation dropped a lot.” These transitional dips call for timely institutional support—such as internship opportunities or research involvement, or curriculum enrichment—to re-establish positive motivational attractors.

Notably, although many universities arrange overseas exchange programs for Thai majors in the first semester of the junior year, our findings suggest that such experiences did not effectively mitigate the motivational decline commonly observed during this period. This pattern contrasts with findings from studies on study-abroad experiences in Western contexts, where learners of major world languages often report motivational gains following immersion abroad (Mori and Gobel, 2021; Yue et al., 2022). Interview data indicate that this discrepancy may be attributed to the limited depth of sociocultural engagement during exchanges to Thailand, as students frequently studied and lived in cohort-based groups with peers from their home institutions, thereby restricting meaningful interaction with local communities. These findings suggest that mobility alone is insufficient to enhance motivation; rather, the quality of sociocultural immersion is crucial. Accordingly, future exchange programs for Thai majors should incorporate more structured opportunities for community engagement—such as internships or sustained interaction with local residents—to support deeper integration and more durable motivational development.

During the final academic year, students' motivational levels displayed abrupt and erratic changes. CDST characterizes such periods—often marked by graduation pressure or future uncertainty—as edge-of-chaos states (Larsen-Freeman and Cameron, 2008), where small perturbations can trigger substantial changes. As one participant described, “In the first semester of senior year, I had to prepare for the postgraduate entrance exam while also trying to work on my thesis. In the second semester, I had to start looking for jobs and attend interviews. With so many things happening at the same time, I often felt overwhelmed, and it was hard to stay focused on studying Thai with the same motivation as before.” These findings suggest that senior-year students are highly susceptible to motivational disruption but also possess reconfiguration potential. As such, targeted interventions—career guidance, thesis scaffolding, or peer mentoring—should be implemented to stabilize and channel motivational dynamics during this critical phase.

### 5.2 Influencing factors

Our study identified a multilayered structure of motivational influences, encompassing macro, exo, proximal, and habitual systems. Unlike traditional models such as Gardner's Socio-Educational Model (Gardner, 1985) or Dörnyei's L2 Motivational Self System (Dörnyei, 2009)—which tend to foreground individual-level constructs—the theoretical model constructed in this study enables a more holistic perspective by acknowledging how environmental, institutional, interpersonal, and intrapersonal systems interact over time. Beyond individual dispositions, we found that participants frequently described external factors—including institutional policies, family contexts, and even sociopolitical events—as exerting substantial influence on learners' motivation. This underscores the importance of situating motivational research within broader contextual frameworks, especially in the case of minoritized or regionally significant languages such as Thai, where learners' trajectories are highly sensitive to dynamic external conditions.

This finding echoes the core CDST principle of nested systems (De Bot et al., 2007; Larsen-Freeman and Cameron, 2008), which posits that learning motivation emerges from the dynamic interplay among multiple interdependent layers. Importantly, our research contributes to CDST by constructing a grounded, empirically driven theoretical model that visually maps these multi-level influences on Thai language learners' motivation. The use of grounded theory allowed us to identify not only the categories of influence but also their interrelations, making the complexity of the motivational system more transparent and analyzable. In doing so, this study extends the applicability of CDST to less commonly taught languages, offering a refined lens for understanding motivational development in linguistically and culturally specific learning contexts.

In addition to the well-documented link between language learning motivation and career prospects, our study identified two macro-level influences—political events and disruptive societal incidents—that have received little attention in previous research. Participants frequently described their motivation as being shaped by high-level diplomatic activities and crises such as the COVID-19 pandemic. Similar findings have been reported in research on Vietnamese majors in China, where students' motivation was also significantly affected by pivotal political events and the pandemic (Ren and Wang, 2025a). Taken together, these results suggest that learners of less commonly taught languages—at least within the Southeast Asian context—may be particularly sensitive to broader geopolitical and societal dynamics.

In addition to macro- and institutional-level influences, our study also highlighted the significant role of family environment in shaping Thai majors' motivation, a factor that has been less emphasized in studies on major world languages. For instance, research on English learning motivation has seldom addressed learners' relationships with family members, as university students often study away from home and their motivational dynamics are more strongly shaped by campus-based contexts (Jiang and Luk, 2016; Yang and Pu, 2022). Similarly, studies on Spanish learning motivation rarely consider family factors, given that learners' experiences are largely embedded within institutional and peer networks (Lu et al., 2019; Veselova et al., 2021). By contrast, our findings suggest that in the context of less commonly taught Southeast Asian languages, family ties can exert a substantial motivational influence in participants' accounts. When learners have family members closely connected to Thailand—for example, relatives working or studying there—their motivation to learn Thai may be significantly enhanced. As one student noted, “My cousin works in Thailand and earns a good salary. Talking to him makes me feel more motivated to study Thai, because I can see its real value.” This illustrates how family-related experiences can transform abstract language learning into tangible life opportunities. It also suggests that motivational research on minoritized languages should give greater attention to family as a contextual variable, and that educators and policymakers may need to design interventions that actively incorporate family networks—for instance, by connecting students with relatives or communities who have real-life experience with the target language and culture.

## 6 Conclusion

This study explored the trajectories and driving forces behind Thai majors' foreign language learning motivation using a qualitative approach grounded in CDST. This study revealed that Thai majors' foreign language learning motivation underwent significant fluctuations over their 4 year university experience, marked by non-linearity, sharp rises and declines, and substantial individual variation. These motivational trajectories were influenced by a constellation of factors operating across multiple levels, including macro-level sociopolitical forces, institutional and familial conditions, classroom interactions, and learners' internal dispositions. The findings highlight the dynamic, context-sensitive, and individualized nature of language learning motivation in less commonly taught languages.

Theoretically, this study contributes to motivation research by proposing a multi-layered, grounded model that visualizes how cross-level factors jointly shape learners' motivation over time. It extends the explanatory power of CDST to the context of less commonly taught languages and highlights the value of combining grounded theory with dynamic systems thinking. Practically, the study offers timely insights for curriculum design, learner support, and policy interventions by revealing when and how students are most vulnerable to motivational decline—and what contextual factors may help restore it.

Several limitations should be acknowledged. First, this study relied on retrospective self-reports, which are subject to recall bias and subjective interpretation; therefore, the influencing factors identified should be understood as participants' perceived motivational mechanisms rather than objectively verified causal effects. Second, the observed motivational changes may also be partially explained by alternative factors such as learner maturation, assessment load, which cannot be fully disentangled within a qualitative retrospective design. Finally, although the sample covered multiple institutions, the relatively small sample size and context-specific focus limit the generalizability of the findings.

## Data Availability

The raw data supporting the conclusions of this article will be made available by the authors, without undue reservation.
